# Statins associate with lower risk of biliary tract cancers: A systematic review and meta‐analysis

**DOI:** 10.1002/cam4.4942

**Published:** 2022-06-13

**Authors:** Ka Shing Cheung, Yan Wang Matthew Yeung, Wing Sum Wong, Bofei Li, Wai Kay Seto, Wai K. Leung

**Affiliations:** ^1^ Department of Medicine, The University of Hong Kong Queen Mary Hospital Hong Kong; ^2^ Department of Medicine The University of Hong Kong‐Shenzhen Hospital Shenzhen China; ^3^ Li Ka Shing Faculty of Medicine The University of Hong Kong Hong Kong; ^4^ Department of Thoracic Surgery, Shanghai Chest Hospital Shanghai Jiao Tong University China

**Keywords:** ampulla of Vater cancer, biliary system, biliary tract, biliary tree, chemoprevention, extrahepatic cholangiocarcinoma, gallbladder cancer, intrahepatic cholangiocarcinoma, liver cancer, statins

## Abstract

**Background:**

Biliary tract cancers (BTCs), encompassing cholangiocarcinoma (CCA), gallbladder (GBC), and ampulla of Vater cancers (AVC), are common hepatobiliary cancer after hepatocellular carcinoma with a high mortality rate. As there is no effective chemopreventive agent to prevent BTCs, this study aimed to explore the role of statins on the risk of BTCs.

**Methods:**

PubMed, Embase, and Cochrane Library from inception until 24 April 2020 were searched according to the Meta‐Analyses of Observational Studies in Epidemiology (MOOSE) guidelines. The adjusted risk ratios (aRRs) of BTCs and individual cancer were pooled using a random‐effects model.

**Results:**

Eight observational studies (3 cohort and 5 case–control studies) were included with 10,485,231 patients. The median age was 68.0 years (IQR: 67.0–71.5) and 48.3% were male. Statins were associated with a lower risk of all BTCs (aRR: 0.67; 95% CI: 0.51–0.87). The pooled aRR for CCA was 0.60 (95% CI: 0.38–0.94) and GBC was 0.78 (95% CI: 0.68–0.90). There was only one study on AVC with aRR of 0.96 (95% CI: 0.66–1.41). The pooled aRR for lipophilic and hydrophilic statins was 0.78 (95% CI: 0.69–0.88) and 0.70 (95% CI: 0.61–0.80), respectively. The effects were attenuated in studies that adjusted for aspirin and/or non‐steroidal anti‐inflammatory drugs (aRR: 0.80, 95% CI: 0.72–0.89) and metformin (aRR: 0.80, 95% CI: 0.72–0.90).

**Conclusions:**

Statins, both lipophilic and hydrophobic, were associated with a lower risk of BTCs, particularly CCA and GBC.

## Graphical abstract

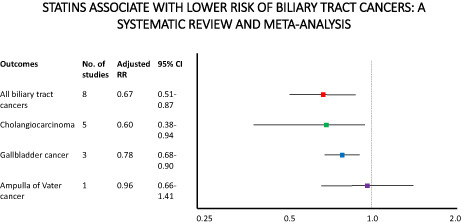

## 1 INTRODUCTION

Biliary tract cancers (BTCs) encompass gallbladder cancer (GBC), extrahepatic cholangiocarcinoma (eCCA), intrahepatic CCA (iCCA), and ampulla of Vater cancer (AVC).^1^ BTCs are the most common hepatobiliary cancer after hepatocellular carcinoma (HCC), with a high mortality rate of 3.58 per 100,000.^1^ There were more than 200,000 new cases of BTCs diagnosed worldwide, with more than 160,000 deaths in 2018.^2^ As the majority of patients present late and at advanced stages, the overall prognosis for BTCs is poor with 5 year mortality of less than 20% for gallbladder cancer,^3^ 20%–30% for eCCA and less than 10% for iCCA.^4^, ^5^, ^6^, ^7^

The risk factors for GBC include cholelithiasis, female sex, and advanced age, whereas risk factors for CCA include diabetes mellitus (DM), smoking, alcohol use, primary sclerosing cholangitis (PSC), liver fluke infestation, nitrosamine exposure, and choledochal cysts.^8^ These risk factors lead to chronic inflammatory process of the biliary tract, which contributes to the pathogenesis of BTCs. Unlike other common malignancies, there is currently no effective prevention and screening program for BTCs, and identification of potential effective chemopreventive agents is eagerly needed. Although meta‐analysis shows that aspirin is associated with a 31% lower risk of CCA,^9^ aspirin can lead to gastrointestinal and intracranial bleeding.^10^ Hence, there is still a need to search for other chemopreventive agents with better safety profiles against BTCs.

Statins, β‐Hydroxy β‐methylglutaryl‐CoA reductase inhibitors, are used in primary and secondary prevention of cardiovascular and cerebrovascular diseases due to its cholesterol‐lowering and anti‐atherosclerotic effects.^11^ The side effects, like hepatotoxicity and myotoxicity, are usually mild and reversible upon cessation of statins. Moreover, studies have shown the pleiotropic effects of statins such as anti‐inflammation and immunomodulation could render potential chemopreventive agents against BTCs.^12^ Statins also have cytotoxic effects on cancer cells.^13^ Molecular studies showed that statins can induce cell apoptosis through superoxide formation, suppression of the mitogen‐activated protein kinase (MAPK) pathway, and reduction of p‐ERK.^14^ Clinical studies showed that statins have chemopreventive effects on various cancers^15^ including gastric cancer^16^ and colorectal cancer.^17^

Recent observational studies on the chemopreventive effects of statins on BTCs are conflicting, particularly their effects on different types of BTCs remain unclear.^18^, ^19^, ^20^, ^21^, ^22^, ^23^, ^24^, ^25^ Herein, we perform a systemic review and meta‐analysis of current literatures on the association between statin use and risks of BTCs.

## 2 METHODS

### 2.1 Study selection

Three databases including Pubmed, Embase, and Web of Science were searched following the MOOSE guidelines^26^ from inception until 24 April 2020. The search details are shown in supplementary file. Key terms included biliary tract cancers (biliary tract tumor, bile duct carcinoma, bile duct neoplasms, cholangiocarcinoma, common bile duct neoplasms, gallbladder cancer) and statins. Manual search for articles through screening of the reference lists of the eligible articles was also performed. Potential studies were retrieved after title/abstract screening by the investigator (MYWY and WSW). All articles were imported to Endnote X9.2 (Thompson and Reuters), and duplicates were removed.

### 2.2 Selection criteria

Two authors (MYWY and WSW) determined the eligibility of studies independently, and discordance was resolved by two senior authors (KSC and WKL). The inclusion criteria included^1^ study population of adult patients (age >18)^2^; statins as the exposure of interest (including lipophilic and hydrophilic statins)^3^; study design of prospective/retrospective cohort study, case–control study, and RCT with adjusted effect estimates provided (adjusted odds ratio [aOR], adjusted hazard ratio [aHR], adjusted relative risk); and^4^ outcomes of BTCs. There was no language restriction. The exclusion criteria were^1^ review articles, editorials, case reports, and other descriptive formats (e.g., commentary and letters)^2^; studies that only reported unadjusted effect estimates; and^3^ studies that reported liver cancer only without stratification according to HCC and iCCA.

### 2.3 Data extraction and quality assessment

For the eligible studies, items including first authors, publication year, country, study design, inclusion/exclusion criteria, exposure of interest, outcome of interest, sample size, age, sex, follow‐up time, and variables considered in the propensity score (PS) analysis or multivariable analysis were recorded. The PS represents the probability of prescribing statins that is dependent on other covariates (e.g., age, sex, etc). PS matching ensures the balance of measured confounding factors between statins and control groups, so that any difference in the outcome (BTCs) would ideally be due to the effect of statin use only. We also recorded data of adjusted effect estimates (aHR/aOR/adjusted relative risk) derived by PS matching or multivariable analysis. The quality of observational studies was assessed by two authors (MYWY and WSW) independently using the Newcastle‐Ottawa scale (NOS) with the highest score of nine points. The risk of bias was categorized into 3 groups: Low risk (7–9 points), moderate risk (4–6 points), and high risk (<4 points).^27^ Studies with a score of at least seven is regarded as having satisfactory quality. Potential time‐related bias (including immortal time bias in cohort study^28^ and time window bias in case control study^29^) that may spuriously augment beneficial effects of statins were also assessed.

### 2.4 Data analysis

All statistical analyses were performed using the R version 3.2.3 (R Foundation for Statistical Computing) statistical software. Continuous variables were expressed as median (interquartile range [IQR]) or mean (±1 standard deviation [SD]). Comparisons of outcome were expressed as adjusted risk ratio (aRR) with 95% confidence interval (95% CI) using the random‐effects model, and were presented as Forest plot. Studies that did not report adjusted effect estimates were excluded from the primary analysis since striking imbalance in baseline characteristics likely existed between statin and statin non‐users. As statins users more often have cardiovascular risk factors including DM, smoking, alcohol use which are also known risk factors for BTCs,^8^ failure to adjust for these comorbidities would likely bias a potentially protective effect of statins on BTCs towards null or even in the opposite direction (i.e., harmful effect).

We used Cochran Q test to detect heterogeneity among studies, with a *p*‐value < 0.1 indicating significant heterogeneity. We calculate *I* ^2^ statistics to measure the proportion of total variation in study estimates attributed to heterogeneity. *I* ^2^ values of <25%, 25%–75%, and >75% indicate low, moderate, and high heterogeneity, respectively.^30^ Publication bias across studies was assessed by visual inspection of funnel plots and Egger's linear regression test.^31^

The effect estimate of dose of statins on the BTC was also calculated. There were two studies^21^, ^23^ that reported dose–response relationship, and cumulative dose for all statins were stratified into quartiles. For this analysis, usage were divided into three groups: (i) Non‐user of statins, (ii) first and second quartiles combined and (iii) third and fourth quartiles combined, as adopted by Peng et al.^23^

The pooled effect estimate from unadjusted RR was also reported. Subgroup analysis was performed according to the class of statins (lipophilic vs. hydrophilic), cancer type, ethnicity (East vs. West), study design (case–control study vs cohort study), presence of adjustment for aspirin and/or non‐steroidal anti‐inflammatory drugs (NSAIDs), metformin, smoking, alcohol use, DM, and PSC. Lipophilic statins that were investigated in various studies included atorvastatin, simvastatin, lovastatin, fluvastatin, while hydrophilic statins included rosuvastatin and pravastatin. Statin users often had underlying cardiovascular diseases and risk factors necessitating concomitant aspirin and/or metformin use, and these two drugs have been reported to be associated with lower BTC risk.^9^, ^32^

## 3 RESULTS

**FIGURE 1:**
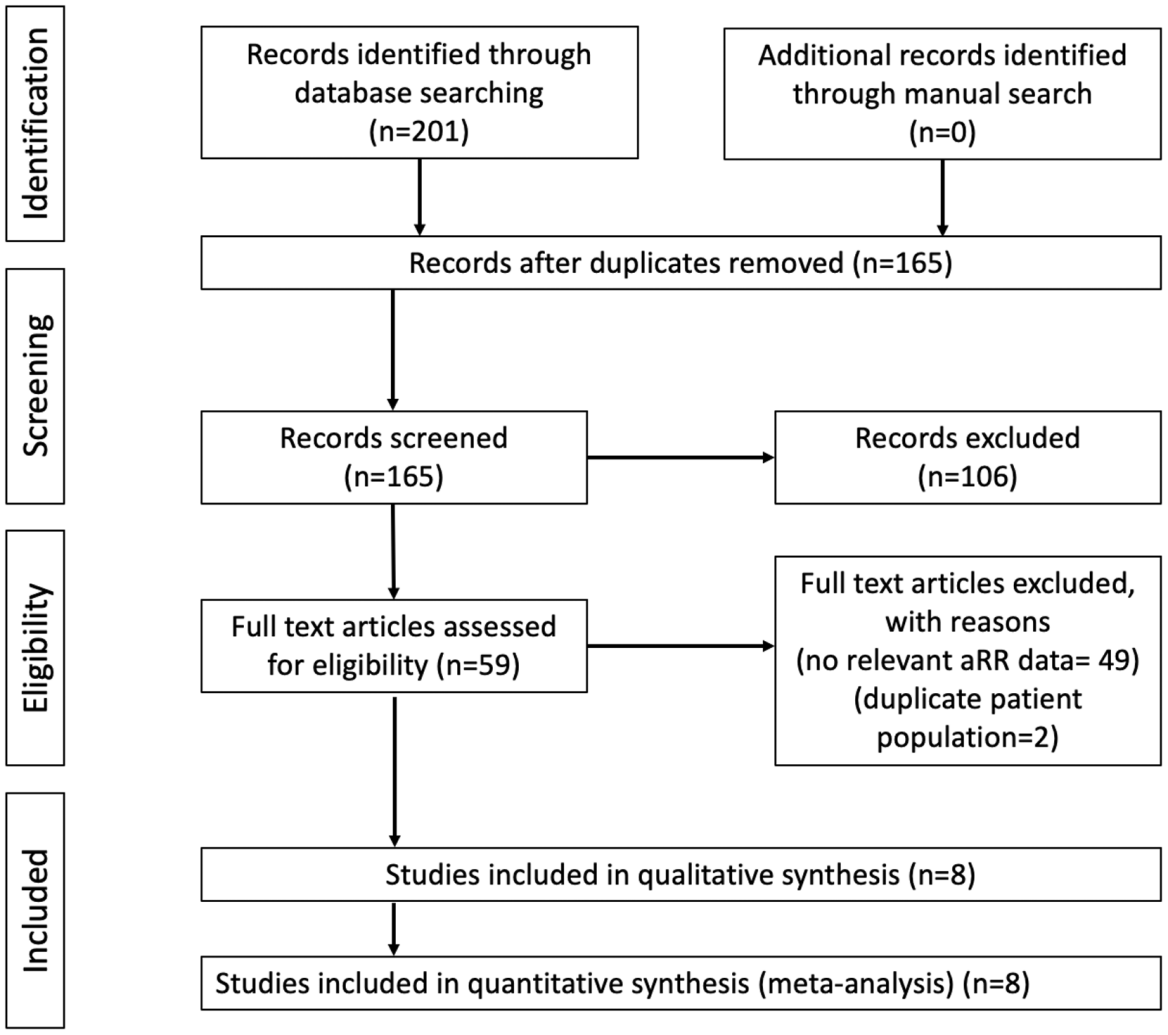
Study selection flow diagram. NOS, Newcastle‐Ottawa scale.

Figure 1 shows the study selection process. Eight out of 201 studies remained in the primary analysis, with two being conference abstracts. All were observational studies with three cohorts and five case–control studies (Table 1). One case–control study reported unadjusted effect estimate only,^33^ and was excluded from the primary analysis. All studies scored at least seven stars in the NOS, indicating satisfactory quality (Table S1). Assessment of time‐related bias is shown: three cohort studies and one case–control study did not have the issue of time‐related bias (Table S2).

**TABLE 1:** Characteristics of the included studies in the meta‐analysis

Author & year	Country (Region)	Study design	Sample Size			BTCs subtype studied	Cancer Prevalence			Statins studied	Adjusted/Matched variables	NOS study quality
			Total	Statin users	Statin non‐users		Total	Statin users	Statin non‐users			
Friedman 2008	USA (West)	Cohort (territory‐wide healthcare delivery program database)	4,222,660	361,859	3,860,801	GBC, eCCA, AVC^b^	22	NS	NS	Lovastatin, Simvastatin, Atorvastatin, Pravastatin, Cerivastatin, Fluvastatin, Rosuvastatin	5	7
Burr 2014	UK (West)	Case control (2 territory hospitals)	356	81	275	CCA	81	16	65	NS	2,7,10,13,15–16	7
Peng 2015	Taiwan (East)	Case control (nationwide NIHRD)	6348	1560	4788	CCA	3174	720	2454	Simvastatin, Lovastatin, Atorvastatin, Pravastatin, Fluvastatin, Rosuvastatin	3,7,9,14,16,19‐29,41–42	8
Marcano‐Bonilla 2018	Sweden (West)	Population‐based cohort (nationwide drug registry database) (CA)	5,760,482	950,635	4,809,847	GBC	1008	NS	NS	NS	NS	8
						iCCA	609	NS	NS			
						eCCA	543	NS	NS			
Liu, Alsaggaf 2019	UK (West)	Case control (nationwide CPRD GOLD database)	18,637	5544	13,093	CCA	1648	511	1167	Atorvastatin, Simvastatin, Pravastatin, Rosuvastatin	10–13	7
						GBC	708	213	495			
						AVC	228	66	162			
Prasai 2019	USA (West)	Case Control (CA)	1899	633	1266	GBC	633	NS	NS	NS	13,15‐17,24,30‐33,41	8
Lavu 2020	USA (West)	Case control (Mayo clinic of Rochester)	1198	482	716	eCCA	412	79	333	NS	4,6,10‐13,16,17,21,24,47,48	8
Tran 2020	UK (West)	Prospective cohort (PCCIU of Scotland, UK biobank of England, Scotland, Wales)	471,851	395,301	76,550	iCCA	72	17	55	Simvastatin, Atorvastatin, Pravastatin, Fluvastatin, Rosuvastatin, Cerivastatin	4,6,8,10‐12,13,16,18,26,30‐31,34‐39,41–46	8
Chaiteerakij 2013^a^	USA (West)	Case control (Mayo clinic of Rochester)	1206	237	969	iCCA	612	72	540	NS	Nil	8

*Note*: 1. age at first prescription; 2. age at diagnosis; 3. age at index date; 4. age at baseline; 5. calendar year; 6. sex; 7. gender; 8. deprivation; 9. Charlson comorbidity index score; 10. smoking; 11. BMI; 12. alcohol drinking; 13. DM; 14. comorbidities of diabetes; 15. PSC; 16. cirrhosis; 17. gallstones; 18. hepatitis; 19. chronic pancreatitis; 20. hepatitis B infection; 21. hepatitis C infection; 22. gastric disease; 23. hemochromatosis; 24. IBD; 25. biliary tract disease; 26. stroke; 27. CAD; 28. COPD; 29. alcohol related illness; 30. hypercholesterolemia; 31. hypertension; 32. hyperthyroidism; 33. hypothyroidism; 34. angina; 35. myocardial infarction; 36. heart failure; 37. peripheral vascular disease; 38. GORD; 39. peptic ulcer disease; 40. interaction between PPIs and statins; 41. aspirin use; 42. metformin use; 43. PPI use; 44. H2RAs use; 45. NSAID use; 46. beta‐blocker use; 47. ethnicity; 48. residence.

Abbreviations: CA, conference abstract; BTCs, biliary tract cancers; CCA, cholangiocarcinoma; iCCA, intrahepatic cholangiocarcinoma; eCCA, extrahepatic cholangiocarcinoma; AVC, ampulla of Vater cancer; GBC, gallbladder cancer; BMI, body mass index; DM, diabetes mellitus; PSC, primary sclerosing cholangitis; IBD, inflammatory bowel disease; CAD, coronary artery disease; COPD, chronic obstructive pulmonary disease; GORD, gastro‐oesophageal reflux disease; PPIs, proton‐pump inhibitors; H2RAs, H2‐receptor antagonists; NS, not specified; NSAIDs, non‐steroidal anti‐inflammatory drugs.

^a^ This study only reported unadjusted risk ratios, and was hence excluded from the primary analysis.

b In this study, the BTC subtypes (GBC, eCCA and AVC) were pooled and analyzed as one single outcome.

### 3.1 Meta‐analysis

A total of 10,485,231 subjects were included in the primary analyses. The median age of patients was 68.0 years (IQR: 67.0–71.5) and 48.3% were male. The characteristics of comparison group are shown in Table S3. Of the eight studies, aHR was reported in three cohort studies and aOR in five case–control studies. Friedmann^24^ reported effect estimates separately for BTCs based on sex, and Marcano‐Bonilla^34^ reported effect estimates separately according to the BTC type (iCCA, eCCA, GBC). Therefore, separate effect estimates were reported in each of these two studies.

**FIGURE 2:**
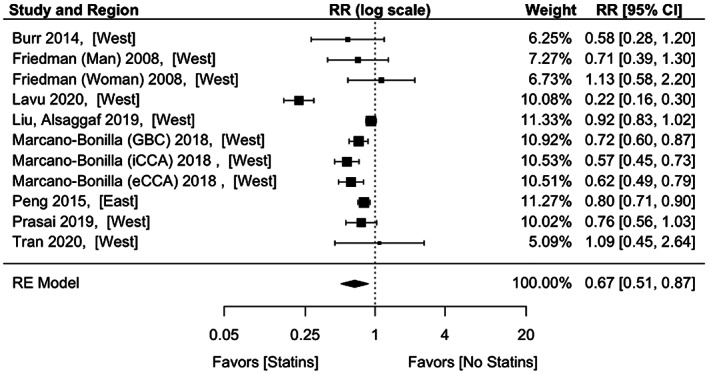
Adjusted effect estimates of statins on biliary tract cancers. RR, risk ratio; RE, random effects; GBC, gallbladder cancer; iCCA, intrahepatic cholangiocarcinoma; eCCA, extrahepatic cholangiocarcinoma.

The pooled aRR of BTCs with all statins was 0.67 (95% CI: 0.51–0.87) (Figure 2). There was significant heterogeneity among the eight studies (*p* < 0.001; *I* ^2^ = 92.46%). Funnel plot did not suggest publication bias (Egger's test: *p* = 0.626) (Figure S1). Five studies reported unadjusted RR, with a pooled value of 0.52 (95% C: 0.32–0.86). There was significant heterogeneity among the studies (*p* < 0.001, *I* ^2^ = 88.72%) (Figure S2).

### 3.2 Effect of individual statin on risk of biliary tract cancer

**FIGURE 3:**
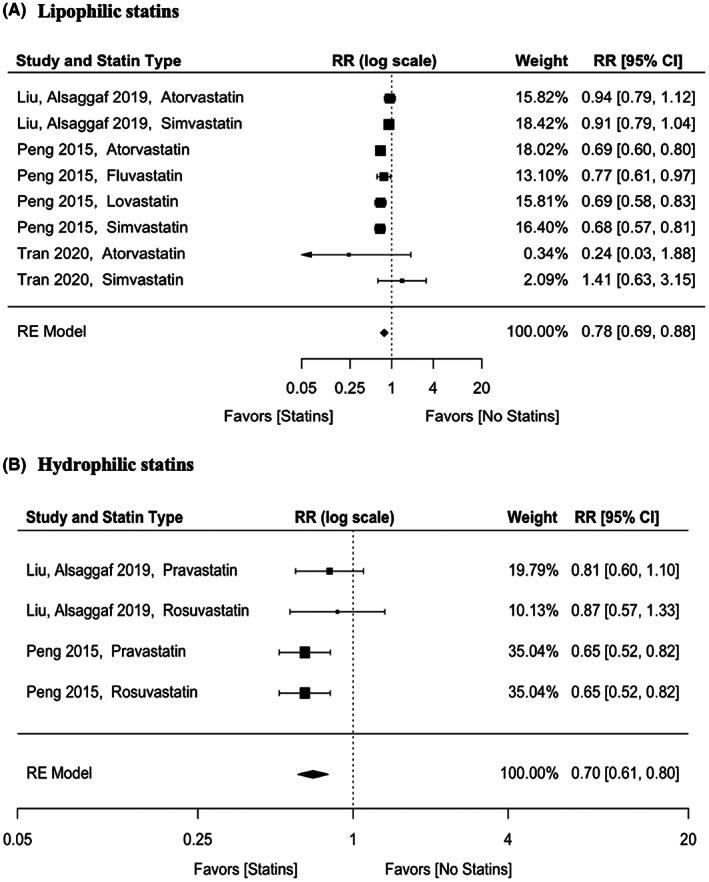
Adjusted effect estimates of statins on biliary tract cancers according to (A) lipophilic statins and (B) hydrophilic statins. RR, risk ratio; RE, random effects.

Three studies included data on the effect of individual statin on BTC risk, including lipophilic and hydrophilic statins (Table S4). For lipophilic statins, the pooled aRR was 0.78 (95% CI: 0.69–0.88) (Figure 3A). There was a moderate level of heterogeneity (*p* = 0.007, *I* ^2^ = 61.15%). Three studies were on atorvastatin with an aRR of 0.77 (95% CI: 0.61–0.98), three on simvastatin with an aRR of 0.85 (95% CI: 0.65–1.13), one on lovastatin with an aRR of 0.69 (95% CI: 0.58–0.83), and one on fluvastatin with an aRR of 0.77 (95% CI: 0.61–0.96) (Table S4). For hydrophilic statins, the pooled aRR was 0.70 (95% CI: 0.61–0.80) (Figure 3B). There was no significant heterogeneity (*p* = 0.438, *I* ^2^ = 0%). There were two studies on rosuvastatin with an aRR of 0.79 (95% CI: 0.53–1.18), and two on pravastatin with an aRR of 0.77 (95% CI: 0.55–1.07) (Table S4).

### 3.3 Dose–response relationship between statins and biliary tract cancer

There were two studies that reported a dose–response relationship between statins and BTCs.^21^, ^23^ Compared with statin non‐use, the aRR was 0.85 (95% CI: 0.77–0.93) for the first and second quartiles combined, and 0.83 (95% CI: 0.76–0.91) for the third and fourth quartiles combined (Table S5).

### 3.4 Subgroup analysis

#### 3.4.1 Cholangiocarcinoma

**FIGURE 4:**
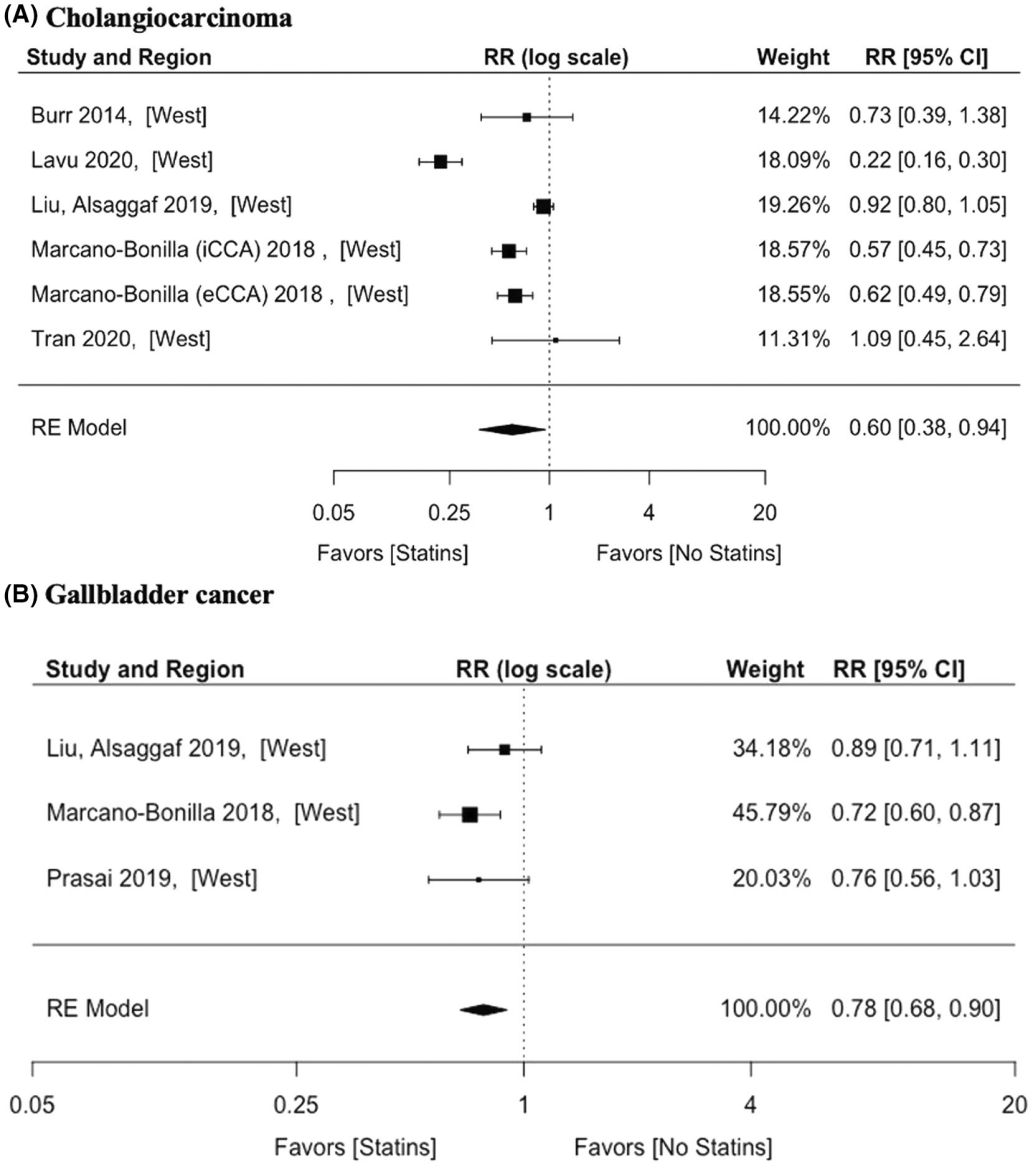
Adjusted effect estimates of statins on (A) cholangiocarcinoma and (B) gallbladder cancer. RR, risk ratio; RE, random effects.

Five studies on CCA were included. The pooled aRR was 0.60 (95% CI: 0.38–0.94), with significant heterogeneity among the studies (*p* < 0.001, *I* ^2^ = 93.52%) (Figure 4A). There were two studies on iCCA, with aHR being 0.68 (95% CI: 0.39–1.20) and significant heterogeneity among the studies (*p* = 0.166, *I* ^2^ = 47.91%). There were two studies on eCCA, with aRR being 0.37 (95% CI: 0.13–1.02) and there was no significant heterogeneity (*p* < 0.001, *I* ^2^ = 96.40%).

#### 3.4.2 Gallbladder cancer

There were three studies investigating GBC, showing an aRR of 0.78 (95% CI: 0.68–0.90). There was no significant heterogeneity between the studies (*p* = 0.353, *I* ^2^ = 16.71%) (Figure 4B).

#### 3.4.3 Ampulla of Vater cancer

There was only one study investigating AVC, reporting an aRR of 0.96 (95% CI: 0.66–1.41).

#### 3.4.4 Study design

**FIGURE 5:**
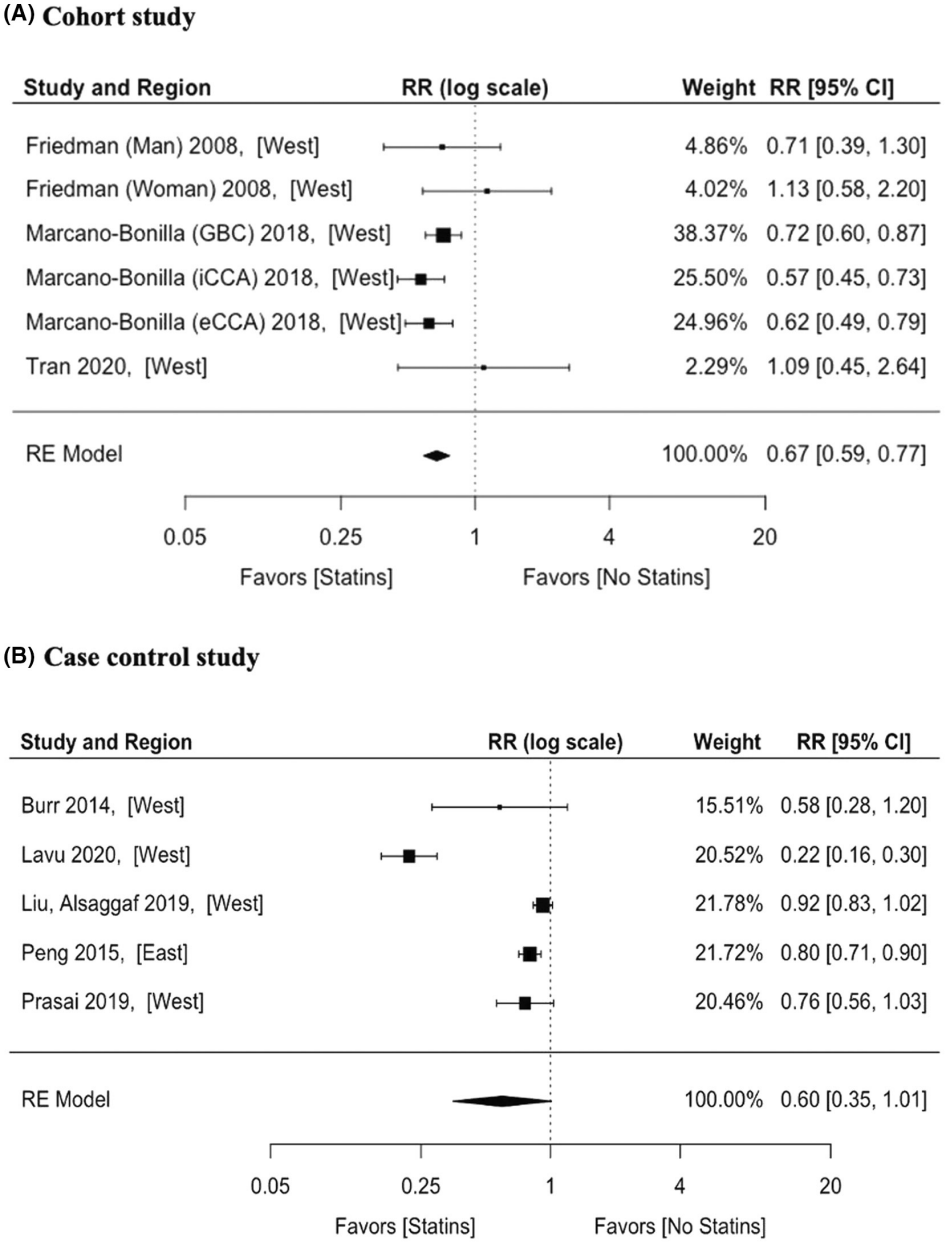
Adjusted effect estimates of statins on biliary tract cancers according to (A) cohort study and (B) case–control study. RR, risk ratio; RE, random effects.

The pooled aHR of the three cohort studies was 0.67 (95% CI: 0.59–0.77) and there was no significant heterogeneity (*p* = 0.282, *I* ^2^ = 11.22%) (Figure 5A). For the five case–control studies, the pooled aOR was 0.60 (95% CI: 0.35–1.01) and there was significant heterogeneity (*p* < 0.001, *I* ^2^ = 97.30%) (Figure 5B).

#### 3.4.5 Geographic region

There were seven studies from the West (United Kingdom = 3, United States of America = 3, Sweden = 1) with pooled aRR of 0.65 (95% CI: 0.48–0.88). There was significant heterogeneity among the studies (*p* < 0.001, *I* ^2^ = 90.96%) (Figure S3). There was only one study from the East (Taiwan) with an aRR of 0.80 (95% C: 0.71–0.90). Notably, this study was the only one that adjusted for infection of liver parasites.

#### 3.4.6 Adjustment for concurrent aspirin/NSAIDs, metformin, smoking, alcohol use, DM, and PSC

Three studies adjusted for aspirin and/or non‐aspirin NSAID use, with a pooled aRR of 0.80 (95% CI: 0.72–0.89) (Figure S4A), while the remaining five studies that did not adjust for aspirin and/or non‐aspirin NSAID use showed a pooled aRR of 0.62 (95% CI: 0.44–0.87) (Figure S4B).

Two studies adjusted for metformin use, with a pooled aRR of 0.80 (95% CI = 0.72–0.90) (Figure S5A). The remaining six studies that did not adjust for metformin use reported a pooled aRR of 0.63 (95% CI: 0.46–0.86) (Figure S5B).

There were five studies that adjusted for smoking with a pooled aRR of 0.62 (95% CI: 0.34–1.11) (Figure S6A). For the three studies that did not adjust for smoking, the pooled aRR was 0.68 (95% CI: 0.60–0.76) (Figure S6B).

Four studies adjusted for alcohol use showed a pooled aRR of 0.63 (95% CI: 0.30–1.30) (Figure S7A), while the remaining four studies that did not adjust for alcohol use showed a pooled aRR of 0.67 (95% CI: 0.60–0.76) (Figure S7B).

Six studies adjusted for DM with a pooled aRR of 0.64 (95% CI: 0.40–1.03) (Figure S8A). For the two studies that did not adjust for DM, the pooled aRR was 0.66 (95% CI: 0.58–0.76) (Figure S8B).

Two studies adjusted for PSC, with a pooled aRR of 0.75 (95% CI: 0.57–0.99) (Figure S9A). The remaining six studies that did not adjust for PSC reported a pooled aRR of 0.67 (95% CI: 0.48–0.92) (Figure S9B).

## 4 DISCUSSION

In this meta‐analysis of eight studies involving more than 10 million patients, we found that statins use was associated with a 33% lower risk of BTCs, including CCA and GBC. Both lipophilic and hydrophobic statins were associated with lower risks of BTCs.

While the main indications of statins are hyperlipidemia and prevention of major vascular events such as stroke or acute coronary syndrome, statins were also found to possess anti‐inflammatory and immunomodulatory effects^12^ that may potentially reduce cancer risk^24^, ^35^, ^36^ in hepatocellular carcinoma, gastric cancer and colorectal cancer.^16^, ^17^, ^37^, ^38^ To our knowledge, this is the first meta‐analysis to demonstrate the chemopreventive effect of statins in BTC. For cancer subtypes, statins were associated with a risk reduction of 40% for CCA and 22% for GBC, respectively. The lack of statistically significant protective effect of statins on subgroup analysis of CCA according to intrahepatic and extrahepatic subsite is likely due to underpower. Similarly for AVC, there was only one study which showed a trend favouring statin use. There may also be a modest trend favouring the dose–response of statins on BTCs prevention, with the lower two and upper two quartiles showing a risk reduction of 15% and 17%, respectively. However, because there were only two available studies investigating the dose–response relationship, more studies are necessary to confirm this observation.

The chemopreventive effects of statins can be explained by the inhibitory effects of statins on cell proliferation via multiple mechanisms. First, statins suppress cell migration and invasion by inhibiting post‐translational modification of G‐proteins that are involved in growth factor signal cascades, cell membrane trafficking, and cell adhesion, such as Ras, Rac, and Rho.^39^ Second, statins induce apoptotic effect via superoxide formation, in which reactive oxygen species and oxidative stress lead to cell dysregulation and induction of the mitochondrial pathway for cell death. The suppression of the MAPK pathway, and thus reduction of p‐ERK, also contributes to cell apoptosis.^14^ Third, given that high cholesterol levels are favourable for cancer growth, disruption of Rac1 activity and thus the colocalization of Rac1 in lipid rafts in cancer cells dampen their proliferation.^40^

Pooling adjusted effect estimates is important in assessing the potential association between statins and cancer development. One may argue that the observed beneficial effect of statins is due to healthy user bias. However, the median age of included subjects in this meta‐analysis was 68.0 years and older individuals (in whom BTCs more likely to occur) generally have more comorbidities that may increase cancer risk, hence potentially negating the concern of healthy user bias.^41^ Nevertheless, in the current meta‐analysis, pooling either adjusted or unadjusted RR yields consistent results (33% and 48% lower risk of BTCs, respectively).

In‐vitro studies suggest a differential chemopreventive effect between hydrophilic and hydrophobic statins—lipophilic statins are associated with cytotoxicity in cancer cells^13^ while hydrophilic statins have no inhibitory effect on HMG‐CoA reductase in extrahepatic cells.^14^

Furthermore, the mechanisms of each type of statin vary. For instance, simvastatin acts on dysregulating the cholesterol biosynthesis pathway, while fluvastatin depends on reactive oxygen species formation. Nevertheless, we found that both lipophilic and hydrophilic statins were associated with a lower risk of BTCs (aRR of 0.78 and 0.70, respectively). Although network meta‐analysis can characterize the comparative efficacy of various statins by indirect comparison, it may not be ideal in this scenario because the scanty number of studies on individual statin effect (*n* = 3). Moreover, observational studies with adjustment for different variables makes it unreliable to compare the effect of individual statins with each other.

We also performed subgroup analysis according to the study design (cohort vs. case–control study), ethnicity (West vs. East), and presence of adjustment for certain variables (including aspirin, metformin, smoking, alcohol use and DM). The pooled effect estimate was similar for cohort and case–control study design (0.67 and 0.60 respectively), although it was of borderline significance for case–control study design. While the pooled effect estimate was 0.65 and 0.80 for studies conducted in West and East respectively, more studies are warranted from the Asian population as there was only one study from Taiwan. The study from Taiwan was the only one that adjusted for parasitic infection. Importantly, the protective effective was lower in studies which adjusted for aspirin (20% vs. 38% risk reduction) and metformin (20% vs. 37% risk reduction). Aspirin reduces CCA risk by suppressing bile duct inflammation and cancer growth factor pathways through COX‐2 inhibition,^9^, ^42^, ^43^ while metformin inhibits CCA tumour growth through cell cycle arrest including targeting the AMPK/mTORC1 pathway.^32^ As a higher proportion of statin users also received aspirin and metformin due to the underlying cardiovascular diseases and risk factors, adjusting for aspirin and metformin use will better delineate the magnitude of protective effect of statins. On the other hand, the pooled aRR was similar after adjusting for smoking, alcohol use and DM (ranging from 0.62 to 0.68). Moreover, a protective effect of statins was demonstrated irrespective of whether PSC had been adjusted for.

A few limitations of this study should be acknowledged. First, there are so far no randomized controlled trials (RCTs) investigating the chemopreventive role of statins in BTCs development. All included studies are observational studies, hence raising the possibility of unmeasured/residual confounding. In particular, case–controls studies generally suffer more biases than cohort studies, including selection bias, recall bias, and interviewer bias. Second, a significant majority of the study subjects were aged ≥60 years, and therefore whether statins are protective among younger subjects requires further investigation. Third, a number of risk factors for BTCs, such as cholelithiasis, PSC, bile duct cysts or parasitic infections,^26^ were not adjusted for in some of these studies. As PSC leads to deranged liver function test, statins may be avoided in PSC, and hence the protective effect may be spuriously augmented without adjusting for PSC. Liver flukes including Opisthorchis viverrini, Clonorchis sinensis, and schistosomiasis japonica are risk factors for biliary tract cancers.^44^, ^45^, ^46^, ^47^ While liver flukes is endemic in Asia, the prevalence in the West is low and hence proportional contribution of liver flukes to biliary tract cancers in the West are unavailable.^47^ Therefore, although parasitic infection was not adjusted for in the studies from the West, its influence on the effect estimates is likely to be low. RCTs, in particular, are desirable to ensure consistency in the eligibility criteria and confounding risk factors that may affect the causal interpretation. Lastly, time‐related bias may exist in observational studies that may spuriously augment the beneficial effects of statins.^28^, ^29^ Nevertheless, three included observational studies that addressed immortal time bias still showed a beneficial effects of statins, although results from case–control studies should be interpreted with more caution as only one of the five included studies addressed time window bias.

## 5 CONCLUSION

Our meta‐analysis showed that statins were associated with a 33% lower risk of BTCs, in particular CCA and GBC. The beneficial effect was observed for both lipophilic and hydrophilic statins, and was consistent with cohort study or case–control study. Future prospective RCTs are warranted to confirm the chemopreventive effect of statins on BTCs development.

## AUTHOR CONTRIBUTION

Ka Shing Cheung was involved with study concept and design, analysis and interpretation of data, drafting of manuscript, and approval of the final version of the manuscript. Matthew YW Yeung and Wing Sum Wong was involved with literature search, analysis and interpretation of data, and drafting of manuscript. Bofei Li was involved with analysis and interpretation of data and drafting of manuscript. WK Seto was involved in interpretation of data and critical revision of the manuscript. Wai K Leung was involved with the study concept and design; analysis and interpretation of data; critical revision of the manuscript for important intellectual content; study supervision; and approval of the final version of the manuscript.

## CONFLICT OF INTEREST

KSC has received speaker fee from AstraZeneca and honorarium for attending advisory board for Janssen and AstraZeneca. WK Seto has received speaker fee from AstraZeneca and Mylan, honorarium for attending advisory board for CSL Behring, and speaker fee and honorarium for attending advisory board for AbbVie and Gilead. WKL has received speaker fee from Eisai, Ferring, Ipsen, and honorarium for attending advisory board for Janssen and Pfizer.

## ETHICS STATEMENT

Ethics approval was not required as this was a meta‐analysis of published articles.

## PATIENT CONSENT STATEMENT

Patient consent was not required as this was a meta‐analysis of published articles.

## Supporting information


Appendix S1

## Data Availability

Data will be shared upon reasonable request.
