# Supplementary material for: Statins associate with lower risk of biliary tract cancers: A systematic review and meta‐analysis
Source: Cancer Med. 2022 Jun 13;12(1):557–68. doi: 10.1002/cam4.4942 (PMC9844660; doi:10.1002/cam4.4942)
Supplement: Supplementary file 1 — Appendix S1 [file CAM4-12-557-s001.pdf]

Tables of Contents

Search details for Embase, Pubmed and Web of Science	pages 2-4
eTable 1. Quality assessment according to Newcastle-Ottawa quality assessment scale (NOS)	page 5
eTable 2. Definition of statin use and risk of time-related bias in the included studies	page 6
eTable 3. Characteristics of comparison groups in the included studies	page 7
eTable 4. Adjusted effect estimates of individual statin on biliary tract cancer risk	page 8
eTable 5. Dose-response relationship of effect of statins on biliary tract cancers	page 9
Figure Legend	page 10
eFigure 1. Funnel plot for detecting publication bias	page 11
eFigure 2. Unadjusted effect estimate of statins on biliary tract cancers	page 12
eFigure 3. Adjusted effect estimate of statins on biliary tract cancers in the West	page 13
eFigure 4. Adjusted effect estimate of statins on biliary tract cancers with stratification according to presence of adjustment for aspirin and/or non-aspirin NSAID use	page 14
eFigure 5. Adjusted effect estimate of statins on biliary tract cancers with stratification according to presence of adjustment for metformin use	page 15
eFigure 6. Adjusted effect estimate of statins on biliary tract cancers with stratification according to presence of adjustment for smoking	Page 16
eFigure 7. Adjusted effect estimate of statins on biliary tract cancers with stratification according to presence of adjustment for alcohol consumption	Page 17
eFigure 8. Adjusted effect estimate of statins on biliary tract cancers with stratification according to presence of adjustment for diabetes mellitus	Page 18
eFigure 9. Adjusted effect estimate of statins on biliary tract cancers with stratification according to presence of adjustment for primary sclerosing cholangitis	Page 19
MOOSE checklist	Pages 20-21

Search details for Embase, Pubmed, and Web of Science

Embase

1. Biliary tract tumor.mp. or exp biliary tract tumor/
2. bile duct carcinoma.mp. or exp bile duct neoplasms/
3. cholangiocarcinoma.mp. or exp cholangiocarcinoma/
4. common bile duct neoplasms.mp. or exp common bile duct neoplasms/
5. Gallbladder cancer.mp. Or exp gallbladder cancer/
6. exp Hydroxymethylglutaryl-CoA Reductase Inhibitors/
7. hydroxymethylglutaryl*.tw.
8. HMG-CoA*.tw.
9. (statin or statins).tw.
10. (atorvastatin or lipitor or cerivastatin or baycol or compactin or fluvastatin or fluindostatin or lescol or lovastatin or mevacor or mevinolin or pitavastatin or pitava or livalo or pravastatin or pravachol or lipostat or rosuvastatin or crestor or simvastatin or zocor).tw.
11. 1 or 2 or 3 or 4 or 5
12. 6 or 7 or 8 or 9 or 10
13. 11 and 12

Pubmed

((((("hydroxymethylglutaryl-coa reductase inhibitors"[Pharmacological Action] OR "hydroxymethylglutaryl-coa reductase inhibitors"[MeSH Terms] OR ("hydroxymethylglutaryl-coa"[All Fields] AND "reductase"[All Fields] AND "inhibitors"[All Fields]) OR "hydroxymethylglutaryl-coa reductase inhibitors"[All Fields] OR ("hydroxymethylglutaryl"[All Fields] AND "coa"[All Fields] AND "reductase"[All Fields] AND "inhibitors"[All Fields]) OR "hydroxymethylglutaryl coa reductase inhibitors"[All Fields]) OR (hydroxymethylglutaryl[All Fields] OR hydroxymethylglutarylation[All Fields] OR hydroxymethylglutarylcoa[All Fields] OR hydroxymethylglutarylcoenzyme[All Fields] OR hydroxymethylglutarylsecoisolariciresinol[All Fields])) OR (hmg coa[All Fields] OR hmg coacetyl[All Fields] OR hmg coar[All Fields] OR hmg coared[All Fields] OR hmg coareductase[All Fields] OR hmg coari[All Fields] OR hmg coas[All Fields] OR hmg coas2[All Fields])) OR (statin[All Fields] OR statin'[All Fields] OR statin's[All Fields] OR statina[All Fields] OR statinadherencia[All Fields] OR statinae[All Fields] OR statinaemics[All Fields] OR statinami[All Fields] OR statinanalogue[All Fields] OR statinanvandning[All Fields] OR statinanwendern[All Fields] OR statinar[All Fields] OR statinary[All Fields] OR statinase[All Fields] OR statinassociated[All Fields] OR statinassocieret[All Fields] OR statinat[All Fields] OR statinated[All Fields] OR statinbehandlas[All Fields] OR statinbehandlede[All Fields] OR statinbehandles[All Fields] OR statinbehandling[All Fields] OR statinbehandlung[All Fields] OR statinbiverkningar[All Fields] OR statinbruk[All Fields] OR statinbytte[All Fields] OR statindosen[All Fields] OR

statindosis[All Fields] OR statine[All Fields] OR statine's[All Fields] OR statinele[All Fields] OR statinem[All Fields] OR statinen[All Fields] OR statiner[All Fields] OR statinergic[All Fields] OR statinernas[All Fields] OR statinernes[All Fields] OR statiners[All Fields] OR statines[All Fields] OR statines'[All Fields] OR statines's[All Fields] OR statinfibrate[All Fields] OR statinflasas[All Fields] OR statinfo[All Fields] OR stating[All Fields] OR statingabe[All Fields] OR statingh[All Fields] OR statini[All Fields] OR statinia[All Fields] OR statinib[All Fields] OR statiniennes[All Fields] OR statinima[All Fields] OR statininduced[All Fields] OR statininducerede[All Fields] OR statininduceret[All Fields] OR statininduzierte[All Fields] OR statininduzierten[All Fields] OR statininduziertes[All Fields] OR statining[All Fields] OR statininhibited[All Fields] OR statinintolerans[All Fields] OR statinintoleranse[All Fields] OR statinintoleranz[All Fields] OR statinization[All Fields] OR statinizing[All Fields] OR statinkezeles[All Fields] OR statinkostnader[All Fields] OR statinler[All Fields] OR statinlerin[All Fields] OR statinlerle[All Fields] OR statinmed[All Fields] OR statinok[All Fields] OR statinokkal[All Fields] OR statinokra[All Fields] OR statinom[All Fields] OR statinoma[All Fields] OR statinopathy[All Fields] OR statinopause[All Fields] OR statinotherapy[All Fields] OR statinov[All Fields] OR statinova[All Fields] OR statinove[All Fields] OR statinovou[All Fields] OR statinpiller[All Fields] OR statinrelated[All Fields] OR statins[All Fields] OR statins'[All Fields] OR statinsat[All Fields] OR statinstudie[All Fields] OR statinstudier[All Fields] OR statinterapia[All Fields] OR statinth[All Fields] OR statintherapie[All Fields] OR statintherapien[All Fields] OR statintherapy[All Fields] OR statintreated[All Fields] OR statintreatment[All Fields] OR statinvalasztas[All Fields] OR statinwirkung[All Fields] OR statinwise[All Fields] OR statiny[All Fields])) OR (("atorvastatin"[MeSH Terms] OR "atorvastatin"[All Fields]) OR ("atorvastatin"[MeSH Terms] OR "atorvastatin"[All Fields] OR "lipitor"[All Fields]) OR ("cerivastatin"[Supplementary Concept] OR "cerivastatin"[All Fields]) OR ("cerivastatin"[Supplementary Concept] OR "cerivastatin"[All Fields] OR "baycol"[All Fields]) OR ("mevastatin"[Supplementary Concept] OR "mevastatin"[All Fields] OR "compactin"[All Fields]) OR ("fluvastatin"[MeSH Terms] OR "fluvastatin"[All Fields]) OR ("fluvastatin"[MeSH Terms] OR "fluvastatin"[All Fields] OR "fluindostatin"[All Fields]) OR ("fluvastatin"[MeSH Terms] OR "fluvastatin"[All Fields] OR "lescol"[All Fields]) OR ("lovastatin"[MeSH Terms] OR "lovastatin"[All Fields]) OR ("lovastatin"[MeSH Terms] OR "lovastatin"[All Fields] OR "mevacor"[All Fields]) OR ("lovastatin"[MeSH Terms] OR "lovastatin"[All Fields] OR "mevinolin"[All Fields]) OR ("pitavastatin"[Supplementary Concept] OR "pitavastatin"[All Fields]) OR pitava[All Fields] OR livalo[All Fields] OR ("pravastatin"[MeSH Terms] OR "pravastatin"[All Fields]) OR ("pravastatin"[MeSH Terms] OR "pravastatin"[All Fields] OR "pravachol"[All Fields]) OR ("pravastatin"[MeSH Terms] OR "pravastatin"[All Fields] OR "lipostat"[All Fields]) OR ("rosuvastatin calcium"[MeSH Terms] OR ("rosuvastatin"[All Fields] AND "calcium"[All Fields]) OR "rosuvastatin calcium"[All Fields] OR "rosuvastatin"[All Fields]) OR ("rosuvastatin calcium"[MeSH Terms] OR ("rosuvastatin"[All Fields] AND "calcium"[All Fields]) OR "rosuvastatin calcium"[All Fields] OR "crestor"[All Fields]) OR ("simvastatin"[MeSH Terms] OR "simvastatin"[All Fields]) OR ("simvastatin"[MeSH Terms] OR "simvastatin"[All Fields] OR "zocor"[All Fields]))) AND ((("biliary tract"[MeSH Terms] OR ("biliary"[All Fields] AND "tract"[All Fields]) OR "biliary tract"[All Fields]) AND ("tumour"[All Fields] OR "neoplasms"[MeSH Terms] OR "neoplasms"[All Fields] OR "tumor"[All Fields])) OR ("bile duct neoplasms"[MeSH Terms] OR ("bile"[All Fields] AND "duct"[All Fields] AND "neoplasms"[All Fields]) OR "bile duct neoplasms"[All Fields] OR ("bile"[All Fields] AND "duct"[All Fields] AND "carcinoma"[All Fields]) OR "bile duct carcinoma"[All Fields]) OR ("bile duct neoplasms"[MeSH Terms] OR ("bile"[All Fields] AND "duct"[All Fields] AND "neoplasms"[All Fields]) OR "bile duct neoplasms"[All

Fields]) OR ("cholangiocarcinoma"[MeSH Terms] OR "cholangiocarcinoma"[All Fields]) OR ("common bile duct neoplasms"[MeSH Terms] OR ("common"[All Fields] AND "bile"[All Fields] AND "duct"[All Fields] AND "neoplasms"[All Fields]) OR "common bile duct neoplasms"[All Fields]) OR ("gallbladder neoplasms"[MeSH Terms] OR ("gallbladder"[All Fields] AND "neoplasms"[All Fields]) OR "gallbladder neoplasms"[All Fields] OR ("gallbladder"[All Fields] AND "cancer"[All Fields]) OR "gallbladder cancer"[All Fields]) OR (("ampulla of vater"[MeSH Terms] OR ("ampulla"[All Fields] AND "vater"[All Fields]) OR "ampulla of vater"[All Fields]) AND ("neoplasms"[MeSH Terms] OR "neoplasms"[All Fields] OR "cancer"[All Fields])))

Web of science

Search terms for statins

- hydroxymethylglutaryl-coa reductase inhibitor OR
- Statin OR
- Atorvastatin OR lipitor OR cerivastatin OR baycol OR mevastatin OR compactin OR fluvastatin OR lescol OR lovastatin OR mevacor OR pitavastatin OR mevinolin OR pravastatin OR pravachol OR lipostat OR rosuvastatin calcium OR crestor oR simvastatin

Search terms for BTC

- Biliary tract tumours
- Biliary tract neoplasms
- Bile duct neoplasms
- Bile duct carcinoma
- Cholangiocarcinoma
- Common bile duct neoplasms
- Gallbladder neoplasms
- Gallbladder cancer
- Ampulla of vater neoplasms
- Ampulla of vater cancer

eTable 1. Quality assessment according to Newcastle-Ottawa quality assessment scale (NOS)

**References**	CA	**Selection**				**Comparability**		**Exposure/Outcome**			**Total**
		Representativeness of exposed cohort	Selection of non-exposed cohort	Ascertainment of exposure	Demonstration that outcome of interest was not present at the start	Controls for age or gender	Controls for additional factor	Assessment of outcome	Follow-up long enough for outcomes to occur	Adequacy of follow-up of cohort	/
Burr 2014	N	1	1	0	1	1	1	1	1	0	7
Chaiteerakij 2013	N	1	1	1	1	1	1	1	1	0	8
Friedman 2008	N	1	1	1	0	0	1	1	1	1	7
Lavu 2020	N	1	1	1	1	1	1	1	1	0	8
Liu, Alsaggaf 2019	N	0	1	1	1	1	1	1	1	0	7
Marcano-Bonilla 2018	Y	1	1	1	1	0	1	1	1	1	8
Peng 2015	N	1	1	1	1	1	1	1	1	0	8
Prasai 2019	Y	1	1	1	1	1	1	1	1	0	8
Tran 2020	N	1	1	0	1	1	1	1	1	1	8

eTable 2. Definition of statin use and risk of time-related bias in the included studies

**Cohort study**		
Study	Definition of statin use and statistical analysis to address time-related bias	Immortal time bias*
Friedman 2008	Treat statin use as time-dependent variable (a subject who once received statins was never later reclassified as a non-user)	No
Marcano Bonilla 2018	Baseline statin use (at least one dispensed prescription)	No
Tran 2020	Baseline statin use	No
**Case control study**		
Study	Definition of statin use and statistical analysis to address time-related bias	Time window bias#
Burr 2014	Statin use ≥ 6 months prior to diagnosis	Yes
Peng 2015	Any statin use prior to diagnosis	Yes
Liu, Alsaggaf 2019	Current user was defined as statin use ending within 2 years prior to the diagnosis/selection date<br><br>Incidence density sampling	No
Prasai 2019	Any statin use prior to diagnosis	Yes
Lavu 2020	Statin use ≥ 3 months prior to diagnosis	Yes

* "immortal time bias" arises if statins users are defined based on their observed duration of statin therapy over follow-up (i.e. postbaseline information to assign baseline treatment status in cohort study.

# "time window bias" arises from the longer time-window for measuring exposure in controls than in cases in case-control studies; to address time-window bias, two methods can be used: either (1) full cohort time-dependent analysis, in which all person-days of follow-up are classed as nonexposed until the first statin prescription, and classified as exposed thereafter; or (2) time-dependent technique based on incidence density sampling, in which random sampling of person-moments of size (e.g. 10 times) the number of cases, selected from all person-moments generated by the cohort.

eTable 3. Characteristics of comparison groups in the included studies

Study	Comparison group
Case control study	
Burr 2014	Patients identified from the dermatology departments in each hospital who had been treated for excision of non-melanotic skin tumors, namely basal cell carcinomas, between 2005 and 2008 in Norwich and in 2007 in Leicester. This control group was used since both cholangiocarcinoma and basal cell carcinomas and are known to be associated with similar age and gender distributions. There is also no known association between basal cell carcinomas and the use of non-steroidal anti-inflammatory drugs, aspirin or statins.
Peng 2015	Control subjects were identified from the Longitudinal Health Insurance Database 2000 (LHID 2000). They were matched on propensity score and diagnosis date in a 1:1 ratio with the cases. LHID is a database containing the claims data from 1996 to 2011 for 1 million people randomly sampled from 2000 National Health Insurance Research Database (NHIRD) enrolment records. The distribution of gender, age and health care costs of the LHID2000 was similar to that of all insured enrollees, as reported by the NHRID in Taiwan. Individuals with previous cancer (ICD-9-CM code 140-208) or incomplete information were excluded.
Liu, Alsaggaf 2019	Five controls per case were randomly selected using incidence density sampling. Controls were individually matched on sex, year of birth (±3 years), diagnosis year (±3 years) and number of years in the general practice and in the UK Clinical Research Practice Link (CPRD) prior to diagnosis/ selection date. All controls were required to be alive, cancer free (except for non-melanoma skin cancer) and have at least 2 years of recorded activity in the CPRD prior to the diagnosis date of their matched case.
Prasai 2019	Controls were matched for age, gender and country of residence from patients who underwent cholecystectomy at Mayo Clinic with patients with gallbladder cancer diagnosed between the years 2000 and 2016 in a 2:1 fashion
Lavu 2020	Control subjects were selected from the Mayo Clinic Biobank, which comprises patients receiving care at the Mayo Clinic who have agreed to participate in this clinic-based database. This database includes a large group of patients seen at the Mayo Clinic and is designed to provide control groups for studies performed at the Mayo Clinic, allowing selection of controls that are matched to cases by age, gender, ethnicity, and residence.
Cohort study	
Friedman 2008	Kaiser Permanente Medical Care Program in northern California (KPMCP). The KPMCP is an integrated prepaid healthcare delivery system that provides comprehensive inpatient and outpatient care, including pharmacy services to over 3 million members, who comprise about 30% of the residents of the areas served surrounding San Francisco Bay and in central California. The membership is fairly representative of the local population except for some under-representation of both extremes of the economic spectrum.
Marcano Bonilla 2018	This cohort includes all adult individuals (≥18 years) residing in Sweden who received at least one dispensed prescription, recorded in the Swedish. Prescribed Drug Registry.
Tran 2020	The UK Biobank is a cohort health resource containing approximately 500,000 volunteer participants aged 40–69 from England, Scotland and Wales recruited from 2006 to 2010.

eTable 4. Adjusted effect estimates of individual statin on biliary tract cancer risk

Statins	Studies (n)	BTC Type	No. of cases	No. of controls	aRR	(95% CI)
**Lipophilic statins**						
Atorvastatin	3	All	719	2349	0.77	0.61-0.98
	3	CCA	415	881	0.78	0.58-1.06
	1	GBC	25	100	0.84	0.63-1.12
	1	AVC	29	126	1.15	0.70-1.87
Simvastatin	3	All	1038	4126	0.85	0.63-1.13
	3	CCA	469	1360	0.84	0.63-1.12
	1	GBC	77	384	0.89	0.70-1.12
	1	AVC	55	258	0.93	0.63-1.39
Lovastatin	1	All	244	337	0.69	0.58-0.83
	1	CCA	244	337	0.69	0.58-0.83
Fluvastatin	1	All	143	183	0.77	0.61-0.96
	1	CCA	143	183	0.77	0.61-0.96
**Hydrophilic statins**						
Rosuvastatin	2	All	198	470	0.79	0.53-1.18
	2	CCA	154	279	0.71	0.55-0.92
	1	GBC	2	25	1.08	0.57-2.02
	1	AVC	6	17	1.73	0.65-4.60
Pravastatin	2	All	242	750	0.77	0.55-1.07
	2	CCA	158	336	0.71	0.57-0.87
	1	GBC	16	59	1.01	0.62-1.65
	1	AVC	10	33	1.64	0.76-3.54
Abbreviations: BTC, biliary tract cancer; aRR, adjusted risk ratio; 95% CI, 95% confidence interval; CCA, cholangiocarcinoma; GBC, gallbladder cancer; AVC, ampulla of Vater cancer						

eTable 5. Dose-response relationship of effect of statins on biliary tract cancers

	aRR	95% CI
Non-Users	Reference	-
Quartile 1 and 2 (Lower Half)	0.85	0.77-0.93
Quartile 3 and 4 (Upper Half)	0.83	0.76-0.91
Abbreviations: BTC, biliary tract cancer; aRR, adjusted risk ratio; 95% CI, 95% confidence interval		

Figure legend

eFigure 1. Funnel plot for detecting publication bias

eFigure 2. Unadjusted effect estimate of statins on biliary tract cancers

Abbreviations: RR, risk ratio; RE, random effects

eFigure 3. Adjusted effect estimate of statins on biliary tract cancers in the West

Abbreviations: RR, risk ratio; RE, random effects

eFigure 4. Adjusted effect estimate of statins on biliary tract cancers with stratification according to presence of adjustment for aspirin use

Abbreviations: RR, risk ratio; RE, random effects

eFigure 5. Adjusted effect estimate of statins on biliary tract cancers with stratification according to presence of adjustment for metformin use

Abbreviations: RR, risk ratio; RE, random effects

eFigure 6. Adjusted effect estimate of statins on biliary tract cancers with stratification according to presence of adjustment for smoking

Abbreviations: RR, risk ratio; RE, random effects

eFigure 7. Adjusted effect estimate of statins on biliary tract cancers with stratification according to presence of adjustment for diabetes mellitus

Abbreviations: RR, risk ratio; RE, random effects

eFigure 8. Adjusted effect estimate of statins on biliary tract cancers with stratification according to presence of adjustment for alcohol consumption

Abbreviations: RR, risk ratio; RE, random effects

eFigure 9. Adjusted effect estimate of statins on biliary tract cancers with stratification according to presence of adjustment for primary sclerosing cholangitis

Abbreviations: RR, risk ratio; RE, random effects

eFigure 1. Funnel plot for detecting publication bias

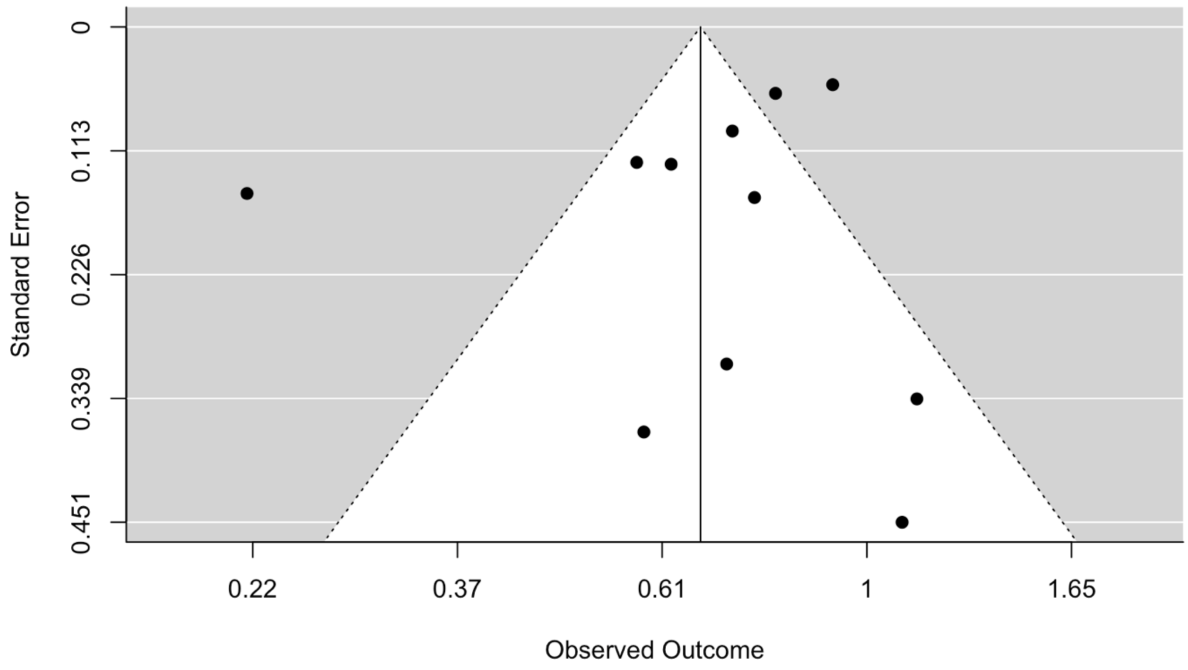

eFigure 2. Unadjusted effect estimate of statins on biliary tract cancers

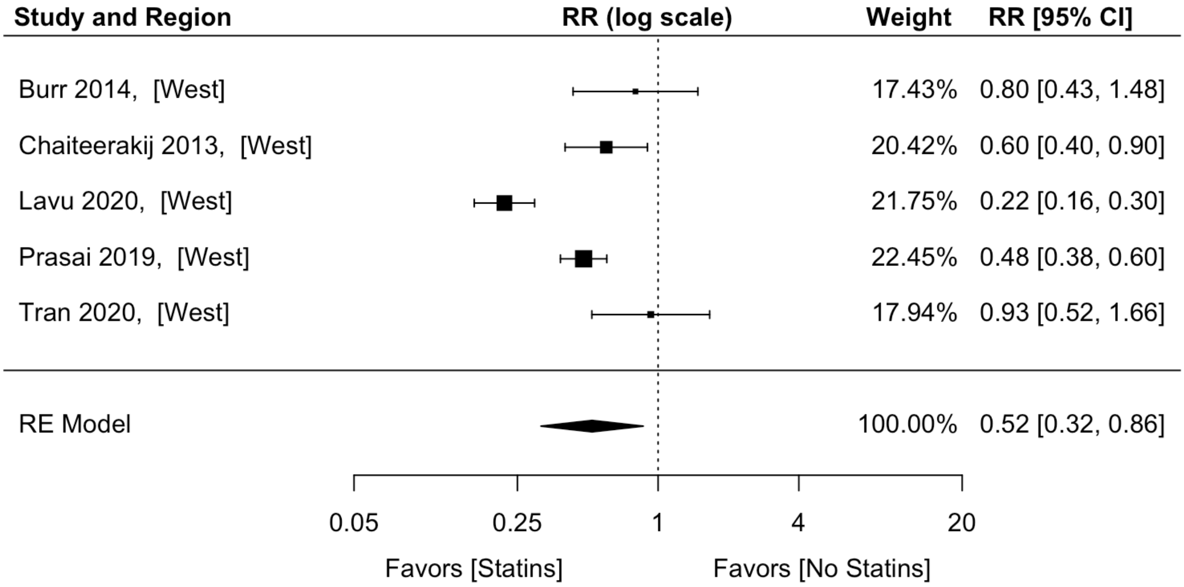

eFigure 3. Adjusted effect estimate of statins on biliary tract cancers in the West

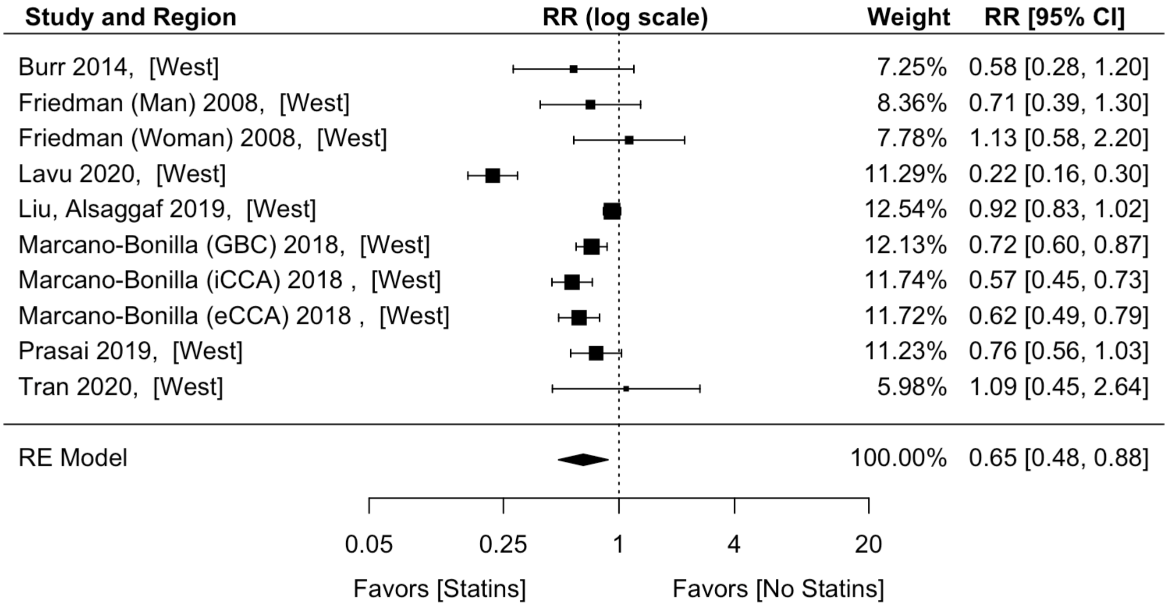

eFigure 4. Adjusted effect estimate of statins on biliary tract cancers with stratification according to adjustment for aspirin and/or non-aspirin NSAID use

(a) Studies adjusting for aspirin and/or non-aspirin NSAID use

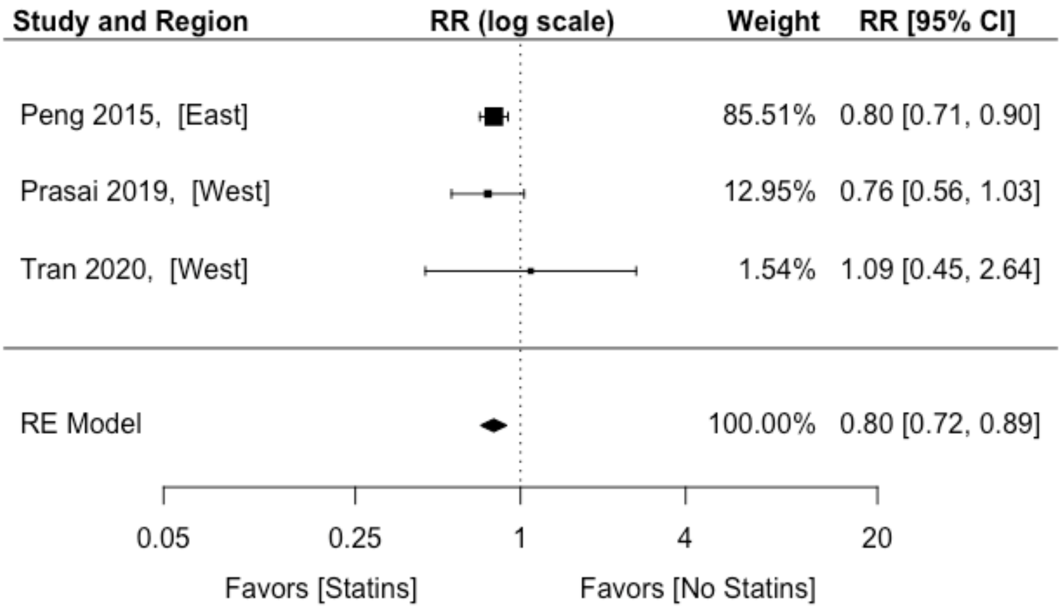

(b) Studies not adjusting for aspirin and/or non-aspirin NSAID use

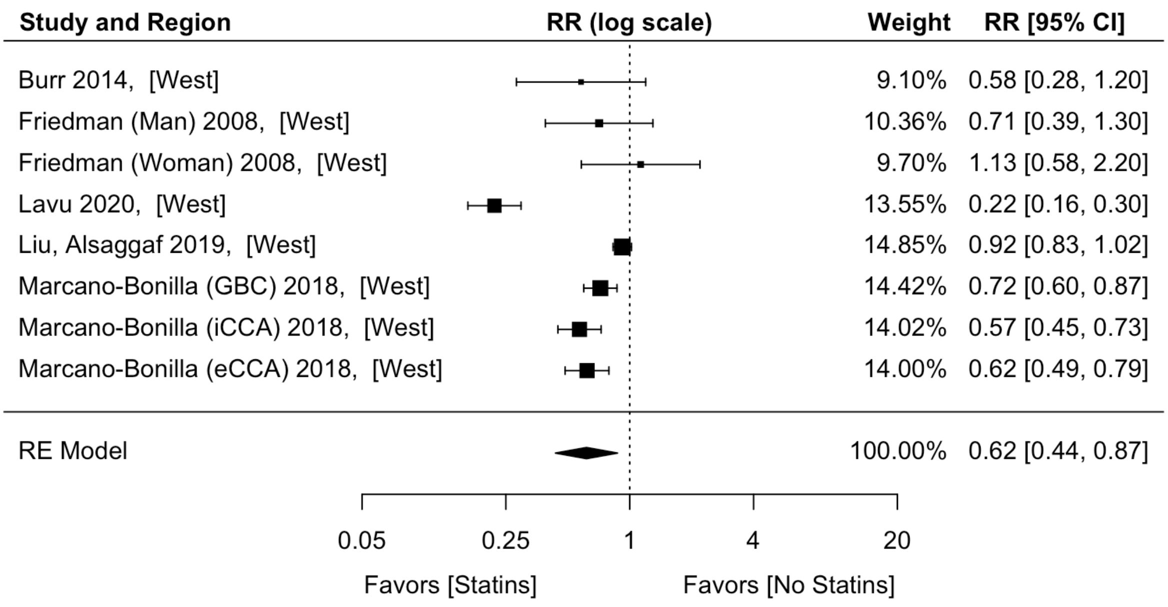

eFigure 5. Adjusted effect estimate of statins on biliary tract cancers with stratification according to adjustment for metformin use

(a) Studies adjusting for metformin use

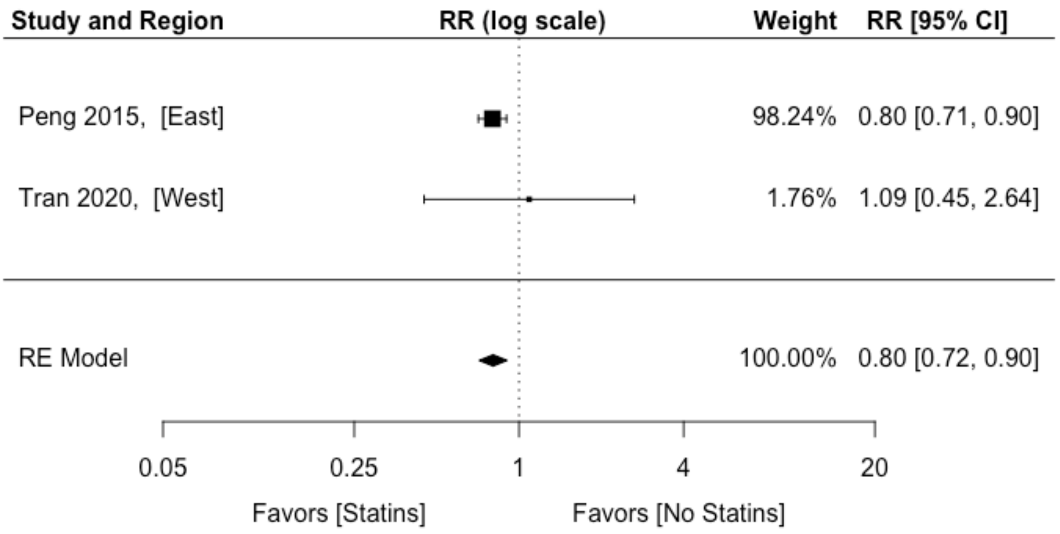

(b) Studies not adjusting for metformin use

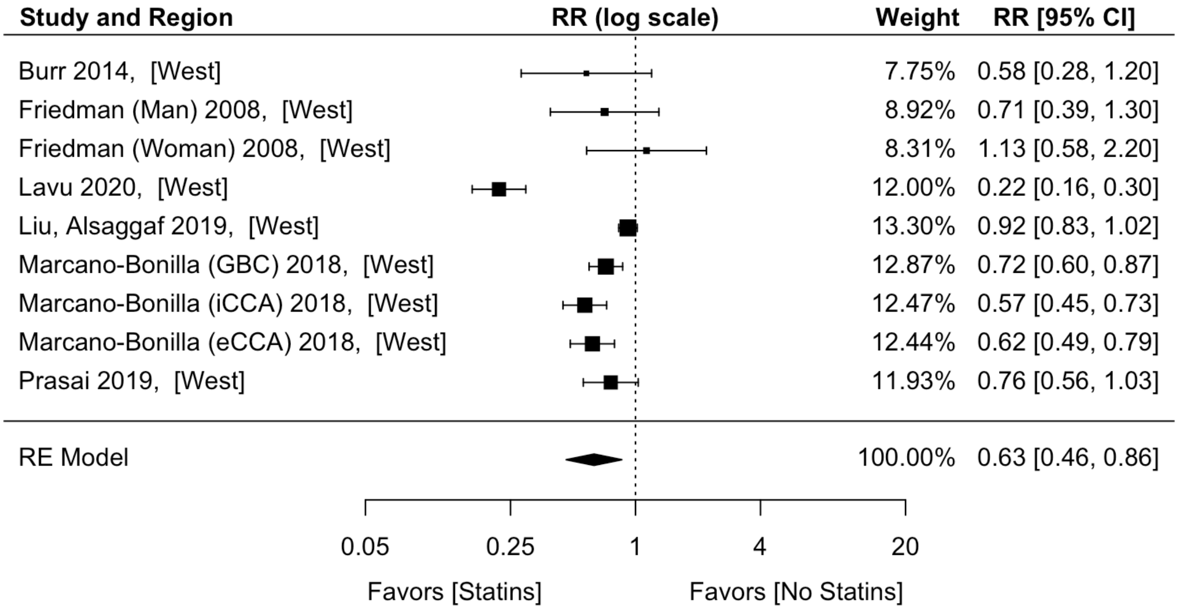

eFigure 6. Adjusted effect estimate of statins on biliary tract cancers with stratification according to adjustment for smoking

(a) Studies adjusting for smoking

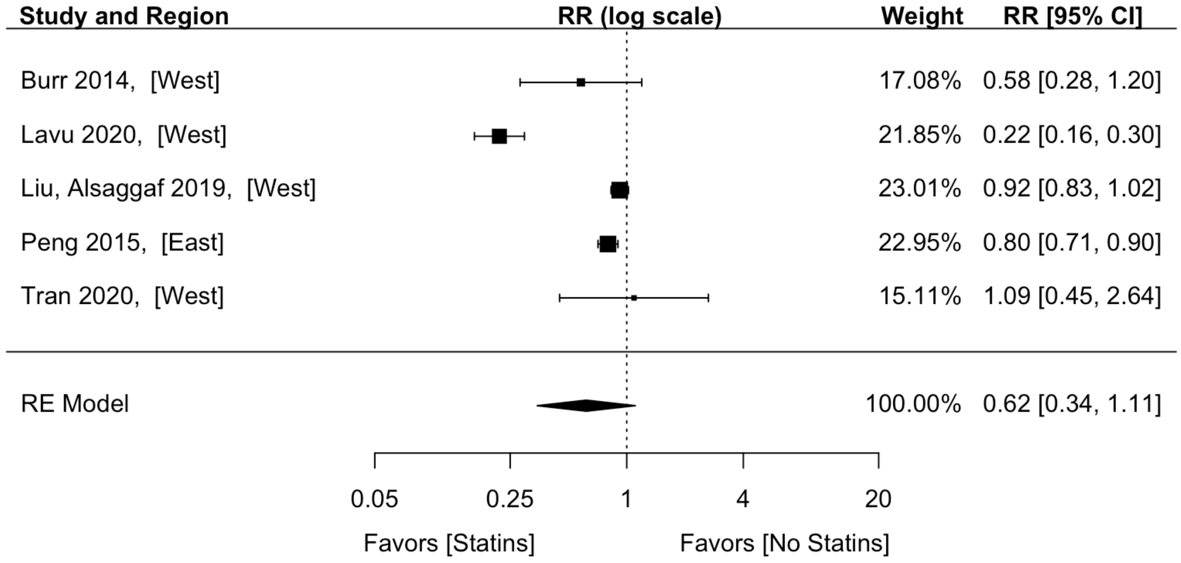

(b) Studies not adjusting for smoking

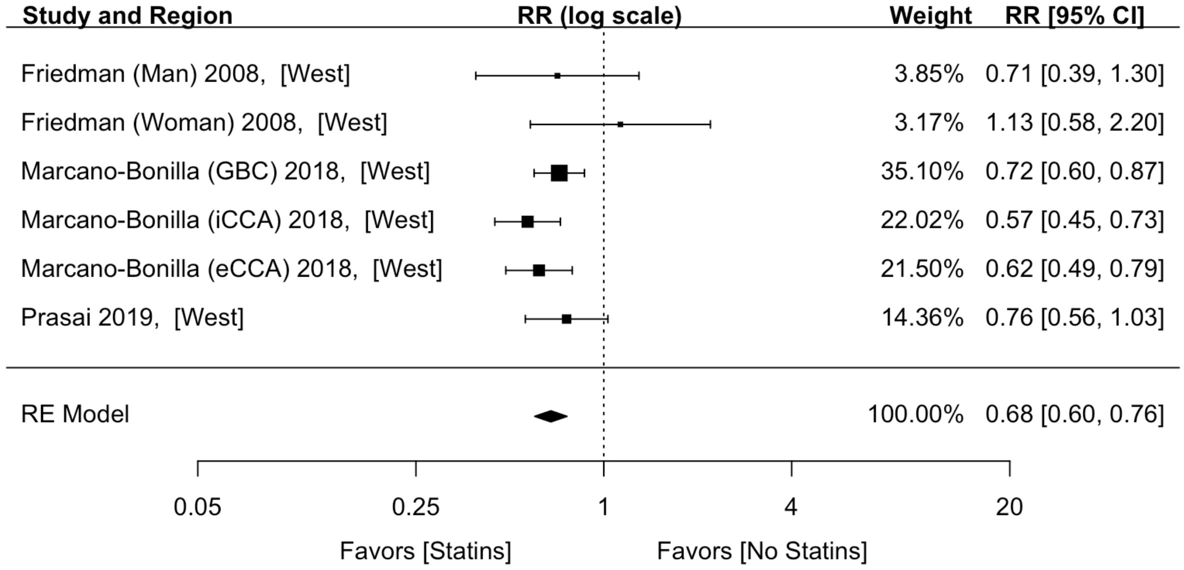

eFigure 7. Adjusted effect estimate of statins on biliary tract cancers with stratification according to adjustment for alcohol consumption

(a) Studies adjusting for alcohol consumption

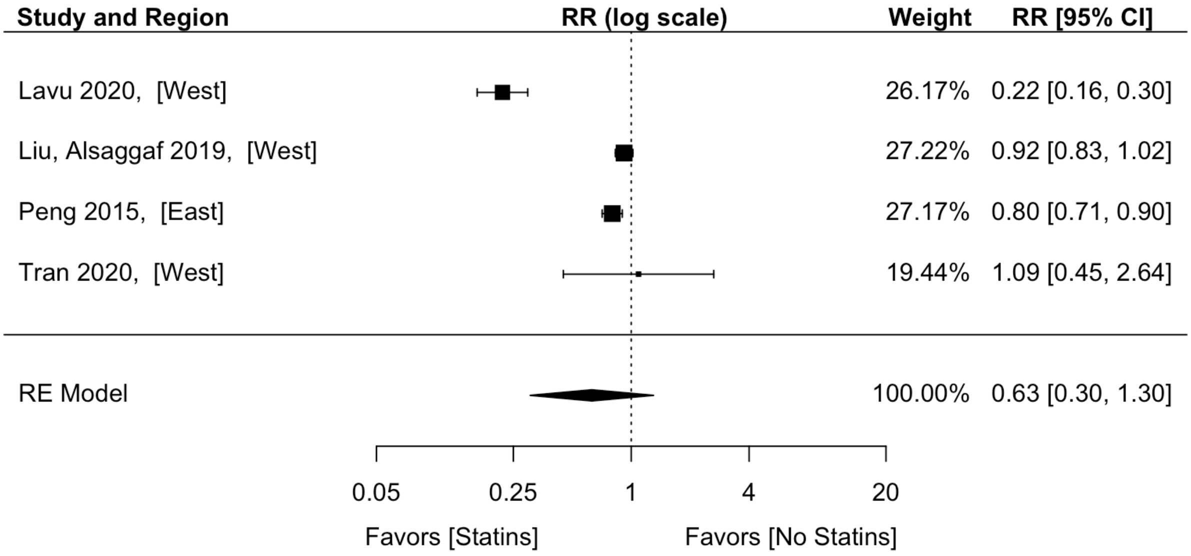

(b) Studies not adjusting for alcohol consumption

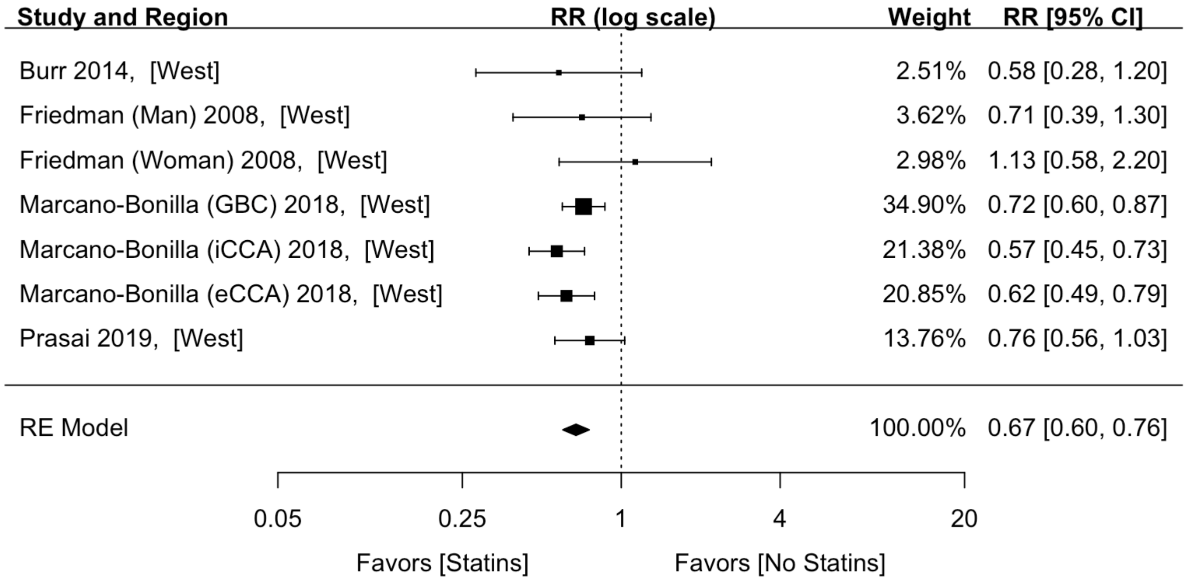

eFigure 8. Adjusted effect estimate of statins on biliary tract cancers with stratification according to adjustment for diabetes mellitus

(a) Studies adjusting for diabetes mellitus

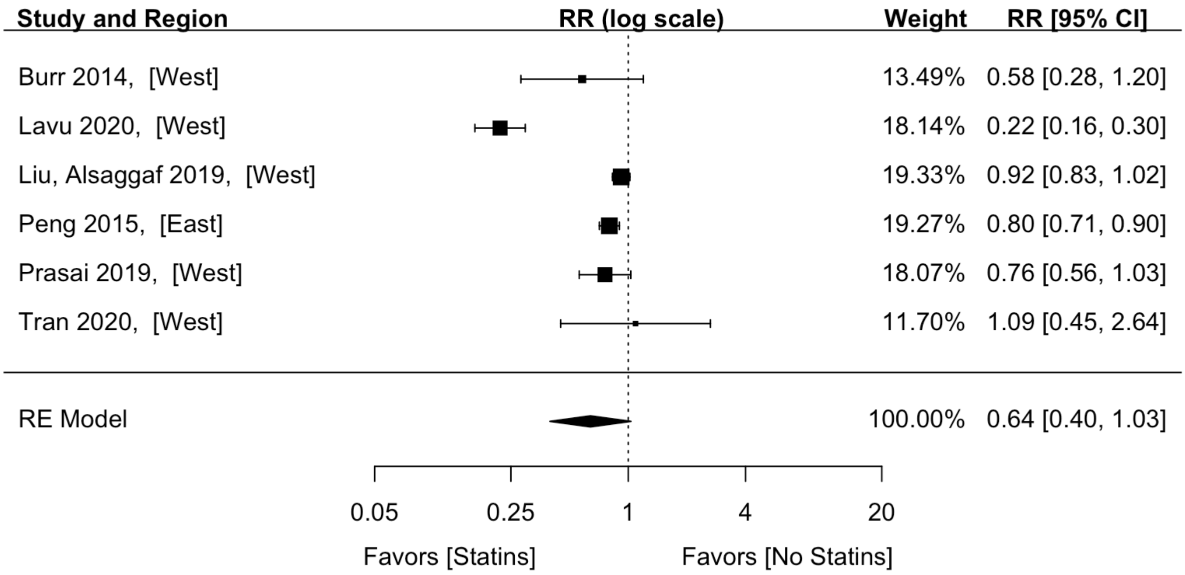

(b) Studies not adjusting for diabetes mellitus

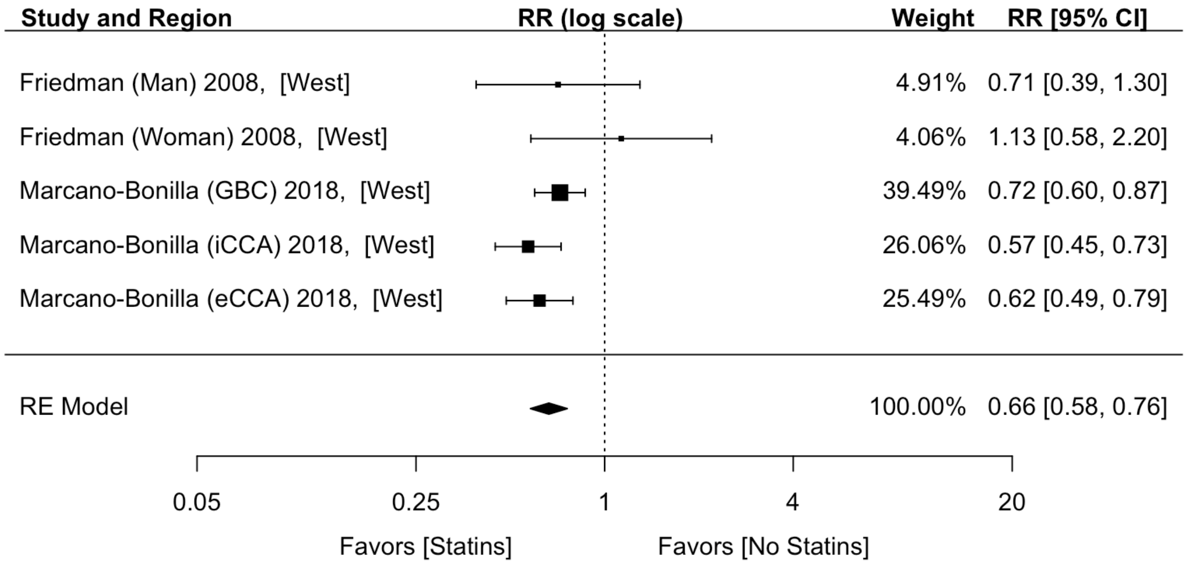

eFigure 9. Adjusted effect estimate of statins on biliary tract cancers with stratification according to adjustment for primary sclerosing cholangitis

(a) Studies adjusting for primary sclerosing cholangitis

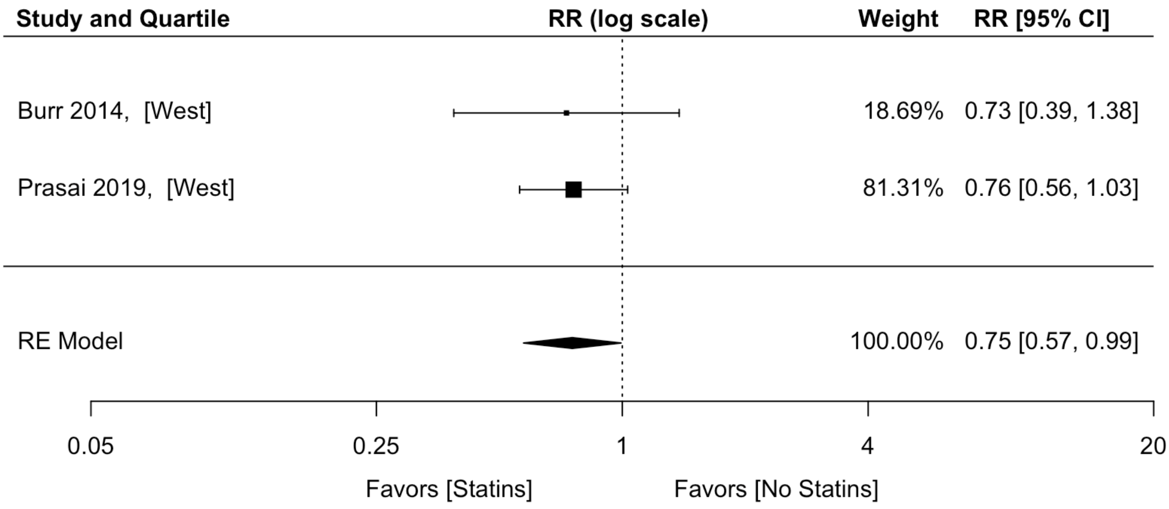

(b) Studies not adjusting for primary sclerosing cholangitis

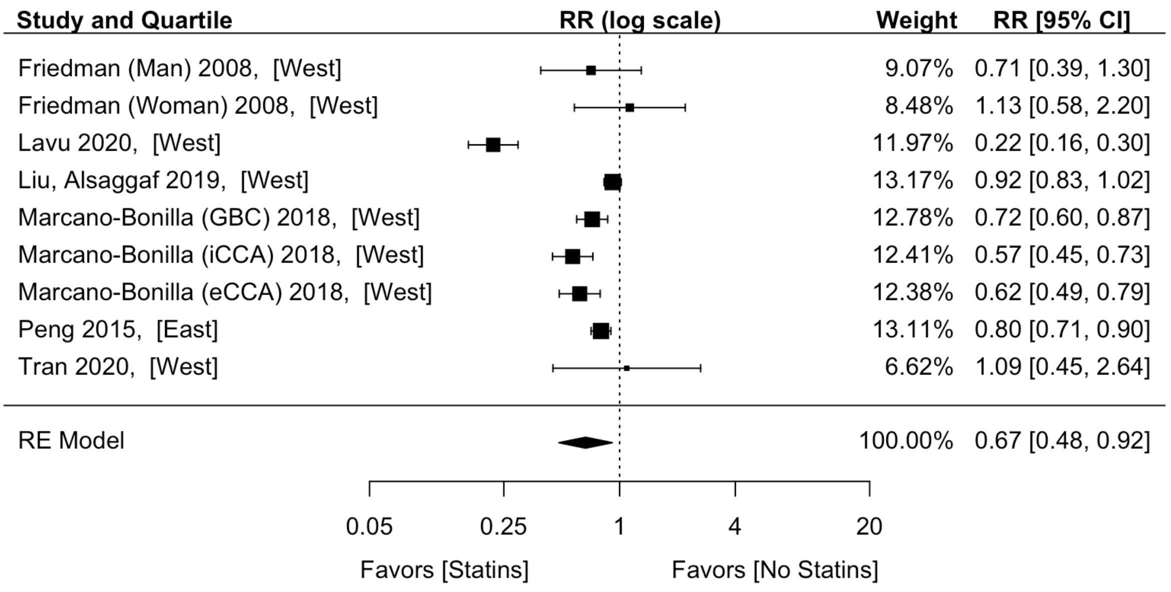

MOOSE Checklist for Meta-analyses of Observational Studies

Item No	Recommendation	Reported on Page No
Reporting of background should include		
1	Problem definition	4
2	Hypothesis statement	4-5
3	Description of study outcome(s)	4-5
4	Type of exposure or intervention used	4-5
5	Type of study designs used	5
6	Study population	5
Reporting of search strategy should include		
7	Qualifications of searchers (eg, librarians and investigators)	1
8	Search strategy, including time period included in the synthesis and key words	6
9	Effort to include all available studies, including contact with authors	6
10	Databases and registries searched	6
11	Search software used, name and version, including special features used (eg, explosion)	6, supplementary file
12	Use of hand searching (eg, reference lists of obtained articles)	6
13	List of citations located and those excluded, including justification	6
14	Method of addressing articles published in languages other than English	6
15	Method of handling abstracts and unpublished studies	6
16	Description of any contact with authors	6
Reporting of methods should include		
17	Description of relevance or appropriateness of studies assembled for assessing the hypothesis to be tested	7
18	Rationale for the selection and coding of data (eg, sound clinical principles or convenience)	7
19	Documentation of how data were classified and coded (eg, multiple raters, blinding and interrater reliability)	7
20	Assessment of confounding (eg, comparability of cases and controls in studies where appropriate)	8
21	Assessment of study quality, including blinding of quality assessors, stratification or regression on possible predictors of study results	7
22	Assessment of heterogeneity	8
23	Description of statistical methods (eg, complete description of fixed or random effects models, justification of whether the chosen models account for predictors of study results, dose-response models, or cumulative meta-analysis) in sufficient detail to be replicated	7-8
24	Provision of appropriate tables and graphics	Figure 1, eTable1
Reporting of results should include		
25	Graphic summarizing individual study estimates and overall estimate	Figure 2
26	Table giving descriptive information for each study included	Table 1

27	Results of sensitivity testing (eg, subgroup analysis)	Figure 3-5, eTable2-3, eFigure2-8
28	Indication of statistical uncertainty of findings	9-12
Reporting of discussion should include		
29	Quantitative assessment of bias (eg, publication bias)	9
30	Justification for exclusion (eg, exclusion of non-English language citations)	9, Figure 1
31	Assessment of quality of included studies	eTable 1
Reporting of conclusions should include		
32	Consideration of alternative explanations for observed results	14-16
33	Generalization of the conclusions (ie, appropriate for the data presented and within the domain of the literature review)	16
34	Guidelines for future research	16
35	Disclosure of funding source	2
